# Gene Therapy and its Implications in Dentistry

**DOI:** 10.5005/jp-journals-10005-1088

**Published:** 2010-04-15

**Authors:** AR Prabhakar, Jibi M Paul, N Basappa

**Affiliations:** 1 Professor and Head, Department of Pedodontics and Preventive Dentistry, Bapuji Dental College and Hospital, Davangere, Karnataka, India; 2 Postgraduate Student, Department of Pedodontics and Preventive Dentistry, Bapuji Dental College and Hospital, Davangere, Karnataka, India; 3 Professor, Department of Pedodontics and Preventive Dentistry, Bapuji Dental College and Hospital, Davangere, Karnataka, India

**Keywords:** Gene therapy, Gene transfer, Bone repair, Salivary glands, Cancer, Pain, Keratinocytes, DNA vaccination, Orthodontic tooth movement.

## Abstract

**Background:**

The concept of transferring genes to tissues for clinical applications has been discussed for nearly half a century. The exponential increase in our ability to manipulate the genetic material of a cell via recombinant DNA technology has brought this goal closer to realization. The original perception that gene therapy should be considered only for a few major organs as a means of treating life-threatening disorders that are refractory to conventional treatment has changed. There are many non-life-threatening conditions that adversely affect a patient’s quality of life, for which there are no effective treatments. The lack of suitable treatment has permitted morbidity to become a rational basis for extending the scope of gene therapy. In the past few years, remarkable progress has been made in the field of gene therapy. While considerable problems remain, thus impeding the routine clinical use of gene transfer, gene therapy will have a pervasive and significant impact on areas that are based on biological science.

**Aim:**

The purpose of this review is to examine the progress made in addressing gene transfer strategies for correcting various diseases and problems that are relevant to dental practice.

## INTRODUCTION

In research facilities all around the world, scientists are attempting to stop diseases at their very roots. Instead of trying to find drugs to cure illnesses they are trying to change the genes that cause the diseases. The process by which this is done is called gene therapy. It is a technique to deliver small DNA or RNA sequences to cells or tissues to correct a genetic defect or treat a disease. The earliest applications of gene therapy were based on the principle that a disease is caused by a faulty gene (or combination of genes) and if such genes can be replaced with ‘correct’ versions, the disease might be controlled, prevented or cured, either *in vivo* or *ex vivo,* and not necessarily a gene that is known to cause a disease. As time passed, new technologies, techniques, strategies and ideas for transferring genes have been presented. Originally known as ‘genetic replacement therapy’ during the early 1980s, ‘gene therapy’ has now outgrown its original definition and is applied to all manner of protocols that involve an element of gene transfer.^1^ It is now widely recognized that gene transfer offers the possibility for ingenious treatments for a host of clinical disorders. Many clinical disciplines that are not normally involved in managing life-threatening conditions are recognizing areas in which gene transfer can be applied. While the development of gene transfer tools is still in its infancy, these variety of applications provide an impressive spectrum of the possible applications of modern biology to dentistry.^2^ Accordingly, this article will review few of the dental applications of gene therapy in some detail.

## WHAT ARE GENES?

Genes are the smallest functional units of the genetic system, which control the development and function of all organisms. A gene is a distinct portion of a cell’s DNA. Genes are mainly concerned with two types of function―determining the structure of the thousands of different proteins that are present in the human body and controlling where, when and in what quantity each protein is made. Proteins are molecules that have different functions in our body. Some form structures of tissues; some are enzymes that control the thousands of chemical reactions that occur in the body. Some proteins stimulate or suppress multiplication of cells.^3^

## GENERAL PRINCIPLES OF GENE TRANSFER

The concept of gene therapy involves the introduction of exogenous genes into somatic cells that form the organs of the body to produce a desired therapeutic effect. The selected DNA fragment must first be cleaved using restriction endonucleases. The next step in successful gene transfer is the preparation of the vector or vehicle used to transfer the genetic material. The vector must first be isolated, purified and cleaved to allow insertion of the DNA fragment. The DNA fragments then must be joined to the cleaved ends of the vector, effectively closing the molecule. This successful insertion of an exogenous DNA molecule into a vector results in a DNA chimera. These vector constructs are the basis of recombinant DNA techniques. The second step involves introduction of the construct into a cell, allowing the production of a line of genetically identical cells containing the DNA sequence introduced by the vector. This allows mass production of cells with a specifically designed genetic make-up.^4^

The ideal vector would have high efficiency (100% cells are transfected), high specificity and low toxicity.^5^ It is highly unlikely, for the foreseeable future, that any single vector type will meet all needs for all tissues; in other words, different vectors will be needed for different clinical applications. Indeed, vector inadequacies are one of this field’s key shortcomings. However, some currently available vectors are quite useful for certain defined conditions, such as adenoviruses for gene therapy of head and neck cancers.^6^

Various vectors used in gene therapy are given in Table 1.

Of all viral vectors currently being studied, adenoviruses and retroviruses are commonly used. These viruses are attenuated to transfect genes, but they cannot replicate or cause infection. Eliminating their ability to replicate through genetic manipulation of the wild type virus eliminates the pathogenecity of virus. Adenovirus-associated virus (AAV), vaccinia virus, lentivirus, herpes simplex virus and many others are currently being extensively studied in the preclinical setting.^5^ Nonviral methods present certain advantages over viral methods, with simple large scale production and low host immunogenicity being just two.^7,23^ Previously, low levels of transfection and expression of the gene held non-viral methods at a disadvantage; however, recent advances in vector technology have yielded molecules and techniques with transfection efficiencies similar to those of viruses.^7^

The clinical application of gene transfer can be accomplished in either of the two ways: *in vivo* or *ex vivo.* During *in vivo* gene transfer, the foreign gene is injected into the patient by viral and nonviral methods. In contrast, an *ex vivo* gene transfer involves a foreign gene transduced into the cells of a tissue biopsy, outside the body, and then resulting genetically modified cells are transplanted back into patient.^3,8^

**Table 1:** **Table 1:** Vectors used in gene therapy^5^

*Viral vectors*		*Nonviral vectors*	
Adenovirus		Lipid complex	
Retrovirus		Liposomes	
Adenovirus-associated virus (AAV)		Peptide/protein	
Lentivirus		Polymers	
Vaccinia virus		Mechanical	
Herpes simplex virus		Electroporation	
		Gene gun	

## AREAS OF IMPACT ON DENTISTRY

### Bone Repair

The development of effective therapies for bone regeneration is one of the most clinically important long-term goals of research in the mineralized tissue field. Bone loss caused by trauma, neoplasia, reconstructive surgery, congenital defects or periodontal disease is a major worldwide health problem. The regeneration of these bone structures poses vastly more complex problems involving specification of three-dimensional shape as well as the type of tissue formed. Yet, it would be enormously useful in the treatment of craniofacial and other bone anomalies, tooth loss, temporo-mandibular and other joint diseases, traumatic amputations and the consequences of tumor resection.^9^

In general, successful bone regeneration rests on the presence of at least four crucial elements, namely osteoin-duction, differentiation of osteoblasts leading to production of osteoid matrix, osteoconduction and mechanical stimulation. Gene therapy may represent an ideal approach towards augmenting bone regeneration as it enhances the first three conditions needed for bone regeneration: Gene therapy can enhance osteoinduction via expression of growth factors, induce osteoblast differentiation and facilitate the production of osteoid matrix and utilize an osteoconductive apparatus. While first conceived as a systemic treatment for hereditary single-gene defects, localized gene therapy is well suited for bone formation because of the ability to deliver genes to a discrete site. In the case of bone regeneration, transient expression is also a desirable benefit and is readily available with existing gene transfer techniques. Thus, gene therapy in bone regeneration has the unique ability to deliver gene products to precise anatomic locations at elevated levels for an extended duration.^10^

The bone morphogenic proteins (BMPs) enable skeletal tissue formation during embryogenesis, growth, adulthood, and healing. Probably BMPs (BMPs 2, 4 and 7) are the only growth factors who can singly induce *de novo* bone formation both *in vitro* and at heterotopic sites.^11^ An investigation at the University of Virginia Medical School demonstrated that it is possible to directly deliver the BMP-2 gene *in vivo* to tissue via an adenoviral vector (vs using *ex vivo* cellular re-engineering), and thus achieve healing of mandibular osseous defects.^6^ In studies of greater therapeutic significance from the Center for Craniofacial Regeneration at the University of Michigan, the biological activity of Ad-BMP7 was examined in two separate ortho-topic regeneration models involving critical-sized defects of calvaria and long bones and a periodontal alveolar defect.^9^ Although individual BMPs can induce bone formation, there is strong evidence that these factors normally work together to induced bone formation. For example, overlapping expression of BMPs 2, 3 a, 4, 7 and 8 is observed at various times during fracture healing.^12^

The delivery of platelet derived growth factor (PDGF) for tissue engineering of periodontal wound has become an active area of interest because of its potent effect on the regeneration of hard and soft tissues. Since the ‘growth arrest specific (gas) gene’ encodes the PDGF receptor, there is a downregulation of PDGF activity leading to transient biological activity and bioavailability of PDGF at the wound site. To overcome this limitation, recently researchers at the University of Michigan have developed an *in vivo* PDGF-A gene transfer through adenovirus vector (Ad-PDGF-A). The bioactive Ad-PDGF-AA protein released induces sustained tyrosine phosphorylation and corrective downregulation of PDGF receptor, which is encoded by “growth arrest specific (gas) gene”. This extends the effect of PDGF on cell signaling, which is critical for cellular proliferation.^8^

Bone sialoprotein (BSP) is a major noncollagenous protein in bone and other mineralized tissues. Cbfal is a *master gene* in osteogenesis and is involved in BSP gene expression, which controls the cell differentiation during bone repair and regeneration. By the *in vivo* delivery of a BSP gene into an osseous defect, it has been shown to regenerate periodontal alveolar bone.^8^

### *Ex vivo* Gene Transfer for Bone Repair

The advantage of an *ex vivo* gene transfer approach is that the surgeon can select specific cells (i.e. bone marrow cells or stem cells) as the cellular delivery vehicle for specific clinical problems. In addition, *ex vivo* strategies have a high efficiency of cell transduction. It is possible to harvest cells from the patient, have a very short period of infection and reimplant the transduced cells at the appropriate anatomic site.^13^ The cells that have received the most interest as a cellular delivery vehicle are mesenchymal stem cells, muscle-derived stem cells, adipose-derived stem cells, buffy coat cells from bone marrow or blood and skin fibroblasts.^14^ Lee et al demonstrated that muscle cells transduced with Ad-BMP-2 could heal critical-sized calvarial defects in SCID mice. The same group of investigators transduced muscle-derived stem cells with retroviruses containing the cDNAs for either Vascular Endothelial Growth Factor (VEGF) or BMP-4 or both BMP-4 and VEGF. More bone was produced in calvarial defects that were treated with muscle-derived stem cells that had been transduced with retroviruses containing both BMP-4 and VEGF genes than when either BMP-4 or VEGF-transduced cells were implanted alone. The results of this study also suggest that the delivery of multiple genes may enhance bone repair.^15^ It has been demonstrated that human adipose tissues contain fibroblast-like cells that can behave in a similar manner to mesenchymal stem cells. When these cells are harvested from adipose tissue and grown in the appropriate media, they can differentiate into bone, cartilage, muscle or fat.^16^ Skin fibroblasts are attractive cellular delivery vehicles because they are easy to harvest and are readily available in all patients. BMP-7-transfected rats fibroblasts have been shown to heal calvarial defects in Lewis rats. One potential problem when using fibroblasts to deliver osteoinductive signals is that fibrous tissue can actually inhibit bone formation and is the predominant tissue type in fracture nonunion sites.^17^ Another strategy that has shown particular promise is the implantation of bone marrow stromal cells (BMSCs) genetically engineered to over express BMP-2 into critical-sized defects.^18^

### Gene Therapeutics to Salivary Gland

Salivary gland destruction occurs as a result of various pathological conditions, such as radiation therapy for head and neck cancer and Sjogren’s syndrome (SS). Accordingly, the development of a novel treatment to restore or regenerate damaged salivary gland tissue was much awaited till the development of gene therapy.^19^ Salivary glands are excellent target sites for gene transfer. They are capable of producing large amounts of proteins;^6^ and are also encapsulated, a circumstance likely to minimize the undesirable access of administered vectors and transgenes to other tissues.^20^ The anatomical structure of the salivary gland, which resembles the many branches and the trunk of a tree, explains that the apical pole of each glandular cell is accessible for gene delivery by a minimally invasive procedure. The opening of the main duct in the oral cavity is canulated and gene delivery vectors, viral or nonviral, are infused by a retrograde injection.^21^

Gene Therapy for Irradiation-induced Hyposalivation

A study demonstrated the potential of gene therapy to correct irradiation-induced salivary hypofunction. An adenovirus-mediated water channel (aquaporin-1, AQP1) gene transfer into irradiated submandibular glands showed increased saliva flow in a rat model. Another study evaluated the efficacy of a single administration of AdhAQP1 to the parotid glands of adult rhesus monkeys. In this study, a single parotid gland of rhesus monkeys was irradiated with a single dose of 10 Gy and AdhAQP1 was administered intraductally at 19 weeks postirradiation and salivary secretion examined 3, 7 and 14 days later. The results, however, were inconsistent and only two of the four AdhAQP1-treated monkeys displayed increased salivary flow rates compared with the animal administered an irrelevant virus.^19-23^ Gene transfer can also be utilized to augment salivary secretions by transferring genes that encode secretory proteins into salivary glands. The proteins are subsequently secreted in an exocrine manner. This was successfully accomplished in animal studies, with the transfer of the human histatin 3 cDNA to rat submandibular glands. Histatin 3, which normally is not secreted in rodent saliva, was secreted at high levels (up to 1 mg/ml) after gene transfer.^22^

Gene Therapy for Sjogren’s Syndrome Impaired Salivary Gland Function

The principal lesion in SS is lymphocytic infiltration in target tissues. Potential target genes in gene therapy for SS-damaged hyposalivation include inflammatory mediators, cytokine inhibitors, apoptotic molecules, cell-cell interaction or intracellular molecules. A recombinant serotype 2 adeno-associated virus encoding the human VIP transgene (rAAV2hVIP) was administered into the submandibular gland of female NOD mice to examine its ability to alter the progressive SS-like dysfunction in NOD mice. While it led to higher salivary flow rates, there were no differences in focus scores or apoptotic rates. In the experimental group, increased expression of VIP in submandibular gland and serum, and a reduction in cytokines IL2, IL10, IL12 (p70), and tumor necrosis factor-alfa in submandibular gland extracts were observed compared with the control vector results. The results indicated that local delivery of rAAV2hVIP can have disease-modifying and immunosuppressive effects in submandibular gland of the NOD mouse model of SS. Furthermore, a key study reported that the treatment of acute and chronic sialadenitis in B6-gld/gld mice with local fasL gene transfer resulted in a significant reduction in the number of inflammatory foci and in the level of tissue destruction in salivary glands.^19^

Gene Transfer to Salivary Glands

It was shown that rat salivary glands, after being administered the rAd5 vector encoding human alfa-1 antitrypsin (hA1AT), were able to secrete the transgene protein into the bloodstreams. This potential was extended in subsequent studies using another rAd5 vector encoding human growth hormone (hGH), also administered to rat salivary glands.^19^

## GENE THERAPY FOR CANCER

Squamous cell cancer of the head and neck (SCCHN) is the sixth most common cancer worldwide, and includes cancer of the oral cavity, pharynx, larynx and paranasal sinuses.^12^ In contrast to cancer in other parts of the body, head and neck cancer is an attractive target for local gene therapy because of its anatomical location. This allows delivery of vectors directly to the desired site with only a small risk of systemic toxicity. Several strategies have been developed for cancer gene therapy, including (1) immunogenic therapy, which involves modulation of immune responses through the transfer of cytokines, immune accessory molecules or tumor antigens; (2) antiangiogenic therapy, which involves the introduction of genes with antiangiogenic properties in a variety of tumor cells; (3) oncolytic virus therapy, which selectively kills tumor cells but not normal cells; (4) gene replacement therapy, which involves the introduction of tumor suppressor genes, such as p53, in cancer cells; and (5) suicide gene therapy, which is used to transduce cancer cells with a gene construct that is able to convert a prodrug into an active drug that is toxic for target cells.^24^ These approaches may converge and can often be used in combination to amplify potential therapeutic effects. To date, vectors based on retroviruses or adenoviruses have been used most frequently in cancer gene therapy.^25^

The incidence of p53 mutations in head and neck cancer is believed to be higher in recurrent disease.^18^ Replacing a mutated p53 gene with a wild-type (normal) p53 gene is a potential approach to head and neck cancer treatment. This approach is limited by the lack of mutated p53 in many tumors and also by the current limitations of vector technology in delivering the gene. In a study of 17 patients with advanced recurrent or refractory unresectable head and neck cancer, treatment with delivery of the p53 gene using an adenoviral vector found only two patients with tumor regression of more than 50%. An additional 17 patients with resectable disease were treated and two remained disease-free for longer than 2 years.^26^

Another tumor suppressor gene that could be replaced in head and neck cancer therapy is p16, since in SCCHN 80 to 90% of cases show p16 inactivation. Loss of p16 expression is secondary to allelic loss of the 9p21 locus and mutation and/or hypermethylation of the gene. Inactivation of p16 is believed to be one of the first steps in head and neck cancer carcinogenesis, and may therefore be an ideal target for gene replacement therapy. Re-expression of p16 in experimental models using viral constructs has the ability to reverse tumor growth and induce apoptosis.^25^ The tumor suppressor genes p16, p21 and Rb are frequently mutated in head and neck cancer, and therefore are potential gene therapy targets.^26^

Conditionally replicating adenoviruses (CRAds) represent a novel class of anticancer agent. These viruses are genetically modified to selectively replicate in tumor cells and to destroy these cells by inducing their lysis.^26^ Over the past decade, several oncolytic viruses have been tested in humans, and although the safety results are encouraging, efficacy as single agents was limited. Possible hurdles include attenuation of the virus caused by genetic engineering of the virus that renders cancer selectivity, host immune responses and lack of understanding of tumor microenvironment. However, H101, an oncolytic adenovirus similar to Onyx-015 (E1B-55K/E3B-deleted), was recently approved by the Chinese government to be used in conjunction with radiation therapy for the treatment of head and neck cancers. This is the first oncolytic virus product approved by a governmental agency for human use.^27^

Further optimization of vectors is essential for improving the clinical efficacy of cancer gene therapy.

### Pain

Managing or eliminating pain is a major part of dental practice. The use of gene transfer technology offers a potentially novel approach to manipulate specific, localized biochemical pathways involved in pain generation. Gene transfer may be particularly useful for managing chronic and intractable pain.^6^ The use of gene transfer in place of drug delivery to achieve the continuous release of short-lived bioactive peptides in or near the spinal dorsal horn underlies the most common strategies for gene therapy of pain. There are two principal models. The first involves intrathecal injection of vectors derived from adenovirus, AAV or lipid encapsulated plasmids. Both have demonstrated robust antiallodynic and antihyperalgesic effects with prolonged effects shown after two injections of a plasmid coding interleukin-10. The cells transduced by vectors injected into the cerebrospinal fluid are known to include resident meningeal cells lining the intrathecal space as well as neurons and glia in spinal parenchyma. In the second approach, neurons of the Dorsal Root Ganglia (DRG) are transduced by injection of herpes simplex virus-based vectors into the skin. These naturally neurotropic vectors are carried by retrograde axonal transport from the skin to the neuronal perikaryon of the DRG. Here, they effect production of inhibitory neurotransmitters 6 or anti-inflammatory peptides to reduce pain in several different chronic pain models.^28^

Researchers at Mount Sinai School of Medicine injected a virus carrying the gene for an endogenous opioid―a chemical naturally produced by the body that has the same effect as opiate painkillers such as morphine―directly into the spinal fluid of rats. The injections were targeted to the dorsal root ganglia in spinal cord, which act as a ‘pain gate’ by intercepting pain signals from the body on their way to the brain. The new technique produced results that lasted as long as three months from a single injection.^29^ There also is a report from Okayama University Dental School in Japan showing the feasibility of direct gene delivery to the articular surface of the temporomandibular joint.^30^ While considerably more research is needed before gene transfer can be tested clinically as a strategy for chronic pain management, the results of these recent studies suggest real promise.^6^

### Gene transfer to Keratinocytes

Epidermal and oral keratinocytes are potential vehicles for gene therapy. Several features of these tissues can be utilized to achieve delivery of therapeutic gene products for local or systemic delivery. These qualities include (1) the presence of stem cells; (2) the cell-, strata- and site-specific regulation of keratinocyte gene expression; (3) tissue accessibility; and (4) secretory capacity. Such features can be exploited by the use of gene therapy strategies to facilitate (1) identification, enrichment, and targeting of stem cells to ensure the continued presence of the transferred gene; (2) high-level and persistent transgene expression using keratinocyte-specific promoters; (3) tissue access needed for culture and grafting for *ex vivo* therapy and direct *in vivo* gene transfer; (4) secretion of transgene product for local or systemic delivery; and (5) monitoring of genetically modified tissue and removal if treatment termination is required. Optimal gene therapy strategies are being tested in a variety of tissues to treat dominant and recessive genetic disorders as well as acquired diseases, such as neoplasia and infectious disease. Since keratinocyte cultures can be manipulated to favor either proliferation or differentiation, growth conditions may be modified and tailored to a particular gene therapy application. Cultured oral keratinocytes have been grafted to oral surgical defects. They persist at these sites and exhibit normal epithelial morphology.^31^ The ability of transduced human keratinocytes to synthesize and secrete biologically active recombinant proteins has been demonstrated. Human growth hormone, apolipoprotein E and the coagulation cascade factor IX are successfully delivered by genetically modified keratinocytes.^32^ At the Department of Oral and Maxillofacial Surgery of Nagoya University Graduate School of Medicine in Japan used a retroviral vector to express factor IX in human oral mucosal keratinocyte cultures.^6^ RB Rutherford and his collegues found that BMP-7-transduced human oral keratinocyte cells (HOKC) has the capacity to form ectopic bone.^33^ Grafting of *in vitro* reconstituted epithelia is routinely used to regenerate epidermis in patients with burn injuries and chronic ulcers. The risk of complications is low, because implants are easily monitored and excised if needed.^33^

For successful keratinocyte gene therapy, stable and long-term gene expression may be achieved through the use of endogenous, keratinocyte-specific promoters and by targeting stem cells. The longevity of genetically altered keratinocytes in epidermal and oral epithelial grafts may be increased by identifying factors which will improve graft survival. Cell-marking studies through which grafted cells can be followed after being genetically marked with a reporter gene may shed light on the fate of grafted cells and on the persistence of expression in these cells *in vivo.* Ongoing work holds promise that it may soon be possible to characterize the phenotype of epithelial stem cells and to target gene delivery to them *in vivo* and *in vitro.* Such technical advances will open the door to clinical trials using keratinocytes to treat disease.^31^

### DNA Vaccination

For many years, dental scientists have tried to use classical vaccination technology to eradicate dental caries or peri-odontal diseases, thus far achieving mixed success. In the last decade, gene transfer research has led to a novel way to achieve vaccination: Directly delivering DNA in a plasmid *vs* the traditional administration of a purified protein or an attenuated microbe.^6^ The ability to induce an immune response to a protein antigen by administration of plasmid DNA encoding the antigen has been successfully demonstrated in animal models. DNA vaccines consist of a eukaryotic expression vector containing a target gene of interest. While DNA vaccination with a single bacterial gene is ostensibly still a subunit approach to vaccination, it is particularly attractive compared to administration of a preformed protein antigen because the immunogen of interest is actively synthesized *in vivo* in transfected cells.^34^ Many studies had reported that mucosal delivery of DNA in liposome and other materials enhanced the mucosal immunity. It has been reported that the plasmid pCIA-P encoding pac gene of *S.mutans* could induce protective anticaries immune responses in rats by targeted salivary gland immunization.^35,36^

Human periodontitis is thought to be initiated by a principal organism called *P. gingivalis.* Two separate rgp-encoding genes (rgpA and rgpB) are located on the chromosome of *P.gingivalis.* rgpA may play a central role in the pathogenesis of periodontal disease via production of pathophysiologically significant proteins. A study demonstrated that immunization of mice with the rgpA DNA vaccine protects against challenge with invasive *P.gingivalis* strain W50 in the mouse lesion model.^34^ A study showed that delivery of the cDNA for the *P.gingivalis* fim-brial protein into murine salivary glands led to the production of secretory immunoglobulin A specific for this microbial protein. This approach could be used to immunize humans against other oral microbes, such as mutans streptococcus.^22^

Although applications of DNA vaccination are in the earliest stages of use with oropharyngeal tissues, it seems reasonable to suggest that these approaches will play a role in future strategies for preventing periodontal diseases and dental caries.^6^

### Gene Therapy for Orthodontic Tooth Movement

Tooth movement depends on the remodeling of alveolar bone, which is controlled by osteoclasts and osteoblasts. These have two different sources: Stromal cells (osteoblasts) and hemopoietic cells (osteoclasts). The formation of mature bone resorbing osteoclasts from hematopoietic precursors requires interaction with cells from the osteoblastic lineage. Periodontal ligament cells or osteoblastic cells are therefore said to be necessary to support osteoclastogenesis. The molecule mediating this interaction is the receptor activator of the NF-kappa B (RANK) ligand, or RANKL. Osteoclastic precursors express RANK, the receptor for RANKL. RANKL is also a ligand for osteoprotegerin (OPG), which is produced by osteoblastic cells or periodontal ligament cells and acts as a decoy receptor for RANKL, preventing RANKL-RANK binding. Excessive OPG expression can thus suppress osteoclastic formation. Two elegant studies by Kanzaki et al have used gene therapy with OPG and RANKL to accelerate and inhibit orthodontic tooth movement in a rat model. Local RANKL gene transfer to the periodontal tissue accelerated orthodontic tooth movement by approximately 150% after 21 days, without eliciting any systemic effects. The authors concluded that “Local RANKL gene transfer might be a useful tool not only for shortening orthodontic treatment, but also for moving an-kylosed teeth where teeth fused to the surrounding bone”. In contrast, local OPG gene transfer inhibited tooth movement by about 50% after 21 days of forced application. Within 40 years, similar procedures may be used by orthodontist to reduce treatment time and improve results.^37^

### Gene Therapy to Grow New Teeth

Dental researchers hope to grow teeth in the laboratory that can be implanted into the mouths of patients who have lost their natural teeth. These would not be living teeth with nerves and blood vessels, but they would be made of the same substances as human teeth. In order to accomplish this, researchers must find the genes responsible for building the 25 major proteins making up tooth structures. In addition, there may be dozens of other genes involved in instructing the body when, how and where to form a particular tooth. There may be as many as 10% of the total number of genes somehow involved in the formation of teeth. The Baylor College of Medicine has found PAX 9, a master gene critical for tooth development. The hope is we will be able to bioengineer human teeth for replacement in the future.^8^

## CONCLUSION

Given the genetic basis for most diseases, instead of contemplating the future of gene therapy, it might be equally interesting to wonder about the future of gene therapy in the context of drug therapy. Although we still consider current gene transfer methods to be fairly primitive, and associated with significant problems, gene therapy’s acceptance as part of the routine clinical armamentarium, at least for some applications (like head and neck cancer), seems very close. Eventually, however, conventional treatments and gene therapies will overlap.
